# Early growth in preterm infants after hospital discharge in rural Kenya: longitudinal study

**DOI:** 10.11604/pamj.2016.24.158.7795

**Published:** 2016-06-22

**Authors:** Diana Mawia Sammy, Margaret Njambi Chege, Jennifer Oyieke

**Affiliations:** 1 School of Nursing Sciences, P.O Box 19676-00202, University of Nairobi, Kenya

**Keywords:** Prematurity, preterm infant, determinants, early growth

## Abstract

**Introduction:**

Prematurity is the single most important cause of mortality during the neonatal period. The early growth of these infants has been shown to be a predictor of their later growth and neurodevelopmental outcomes. The objective of this study was to establish the determinants of early growth in preterm infants after hospital discharge at the Kitui District Hospital, Kenya.

**Methods:**

A short longitudinal study design was adopted to execute the study. During the period of April and June 2014, all the preterm infants who were discharged from the Kitui District Hospital Newborn Unit were enrolled in the study by obtaining written informed consent from their guardians. The anthropometric measurements of these infants were taken at discharge and repeated two weeks later at the Pediatric Outpatient Clinic and the Maternal Child health Clinic. A questionnaire guided interview was held with the guardians to establish infant and maternal characteristics which influenced the infants' early growth.

**Results:**

A total of 112 participants were enrolled for the study with 106 (94.4%) of them being available for reassessment after two weeks. Majority (72.6%) had deficit in growth by failing to attain the recommended WHO average weight gain of 15g/kg/day. Most of the mothers (63.4%) were between the ages of 20-29 years with half of them being first time mothers. Many of them (66.1%) had only attained primary education and were married (66.1%) to self-employed husbands (56%).

**Conclusion:**

Most of the preterm infants at discharge were females who were born between 33 and 36 weeks gestation. Growth deficit was present in the majority and gestational age at birth was a major determinant of the early growth in these preterm infants.

## Introduction

Every year, an estimated 15 million babies are born prematurely. This translates to more than one baby in every 10 babies delivered and accounts for more than 1.1 million deaths annually [1] Sub Saharan Africa is one of the leading regions with high preterm births at 12.5% which is equally high in Kenya at 12% [2]. These infants are more likely to suffer from both short and long term complications of prematurity such as growth deficit. Assessment of growth in the preterm infants in the neonatal period is based on changes in their anthropometric measurements. Consistence in their weight gain demonstrates optimal growth and is a valuable indicator of their growth and progress. However, few studies have been carried out in sub Saharan Africa during the postnatal period of these infants [3]. In Kenya, prematurity contributes to up to 60% of the infant mortality and it has been identified as one of the indicators towards the achievement of the Millennium Development goal IV [4]. In Kenyatta National Hospital, which is a tertiary referral hospital, a study carried out established that early growth of very low birth weight infants was less than expected by their expected date of delivery [5]. In the Kenya demographic health survey, maternal characteristics and the socio-economic status of the population have been identified as one of the major determinants of the mortality and outcomes of the preterm infants [4]. Prior studies have shown that limiting of early growth failure by monitoring of preterm infants up to two years corrected gestational age promotes better outcomes [6]. Optimal early growth is associated with better later growth and developmental outcomes. The extremely preterm infants are at a greater risk of developing complications of prematurity such as growth deficit. Growth of these infants is influenced by different biological and environmental factors in both mother and the infant [7].

## Methods

**Study setting:** The study was carried out at the Kitui District Hospital, a public health facility in rural Kenya. This is the county referral hospital which serves the former Kitui and Mwingi districts. It has a bed capacity of 200 beds and 44 cots. It is also a teaching hospital for medical officer and nursing officer interns as well as the University of Nairobi and the Kenya Medical Training College students. The hospital newborn unit has a capacity of 15 infants but mostly it accommodates about 20 babies and offers excellent care.

**Study design:** In this longitudinal observational study, a total of 112 preterm infants who were discharged from the hospital newborn unit between April and June 2014 were enrolled for the study. They were then followed two weeks post discharge. Any infant who had congenital anomaly which was likely to interfere with their growth or their guardians declined to participate in the study were excluded.

**Data collection:** Data was obtained from the assessment of the infants' anthropometric measurements which included their body weight, body length and head circumference. A questionnaire guided interview was also held with the guardians to establish the infants' birth history, hospitalization and feeding history as well as the maternal demographics, pregnancy history, their socio-economic characteristics and knowledge and practice of the care of the small baby.

**Procedures:** The infants had their body weight taken using an infant weighing scale to the nearest 50g, body length using an infant meter to the nearest 5 mm and head circumference by use of a tape measure to the nearest 1 mm. The same measurements were repeated again at two weeks post discharge at the Pediatric Outpatient Clinic or Maternal Child Health clinic. A 15-20 minutes questionnaire guided interview was held with the infants guardian at discharge and with the use of a code the infants road to health booklet was labelled for easy follow up.

**Data analysis:** The sample size was calculated with the help of Cochrane's modification of Fishers et al formula. Data was entered using MS Excel and later exported to STATA version 10 Texas for analysis. Descriptive statistics was done using means, medians, ranges and proportions. Anthropometric measurements were analyzed using Mann Whitney's test. Bivariate regression was done by use of the Odd's ratio to test for any associations between the early growth and the infant and maternal characteristics.

**Ethical considerations:** The study was approved by the Kenyatta National Hospital-University of Nairobi (KNH-UON) ethics and research committee and data collection was authorized by the Kitui District Hospital administration. Study participants gave informed consent after being given a detailed explanation about the purpose of the study as well as their right to withdraw any time during the study period. Participants were identified by use of codes and the data was analyzed and presented anonymously.

**Study limitations:** The study period was three months which were too short for further follow up of the infants that could have enabled the researcher to establish the later growth or outcomes of the preterm infants.

## Results

A total of 115 preterm infants were screened for the study. One was excluded because of congenital anomaly and two guardians declined to participate. Of the 112 participants enrolled in to the study at discharge from the Kitui District Hospital Newborn Unit, 106 (94.4%) of them were available for reassessment after two weeks. The six (5.6%) participants who were not reassessed at follow-up were due to two (1.8%) neonatal deaths and four (3.6 %) losses to follow-up. Majority of the infants were females (60%) born at gestational age of 33-36 weeks (54.5%). Most (83.9%) of these infants were born in a health facility through spontaneous vertex delivery (77%). They had an APGAR score of more than 7 in one minute (87.5%) and were not resuscitated at birth (74%). The infant length of hospital stay varied from two to 30 days with only 10% having had to stay for more than a month. Most of them were feeding on breast milk only (92.9%) and they had not suffered any illness post discharge (88.3%).

**Figure 1:**
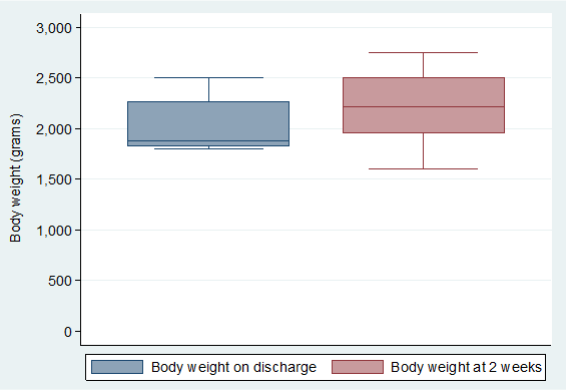
Changes in body weight in preterm infants after hospital discharge at KDH

**Figure 2:**
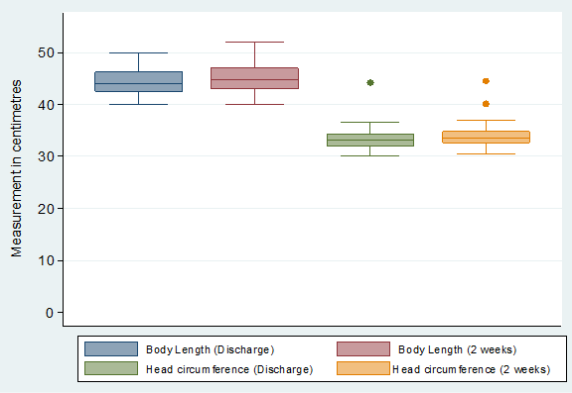
Changes in body length and head circumference in preterm infants after hospital discharge

Most of the mothers were between 20-29 years of age (73.9%) and were married (66.1%). Many of these mothers had only attained primary education (66.1%) and were housewives (35%) to self-employed spouses (56%). Majority of the mothers (88%) lived in the rural areas. The infants' body weight between NBU discharge and week 2 follow-up increased from median body weights of 1880 grams (IQR 1830-2270) and 2215 grams (IQR 1950-2500), respectively (p value < 0.001) (Figure 1). Their median body length increased from 44 cm (IQR 42.5-46.3) to 44.8cm (IQR 43-47), p < 0.001 as shown in Figure 2. Their median head circumference increased from 33.2 cm (IQR 32-34.3) to 33.5 cm (IQR 32.6 -34.8), p < 0.001(Figure 2). There was a statistically significant association between the infants' gestational age at birth and deficit in growth. Infants delivered during 33-36 weeks of gestation had higher odds (OR = 2.97, 95% CI 1.05-9.21) of growth deficits compared to infants delivered at 28-32 weeks (Table 1). There was no statistically significant association between the infant gender, length of hospital stay and maternal characteristics to the early growth in these preterm infants after hospital discharge (Table 2).

**Table 1:** Association between early growth and infant demographics

	Early growth of preterm infants (n = 106)		OR	95% Confidence Interval		P value
	Deficit	Optimal				
**Gender**						
Male	8(27.6)	34(44.2)	1.00			
Female	21(72.4)	43(55.8)	2.08	0.82	5.26	0.124
**Gestation age at birth**						
< 28 weeks	0(0.0)	5(6.5)	NA	NA	NA	NA
28-32 weeks	7(24.1)	35(45.5)	1.00			
33-36 weeks	22(75.9)	37(48.1)	2.97	1.05	9.21	**0.024**
**Mode of delivery**						
SVD	26(89.7)	55(71.4)	1.00			
CS	3(10.3)	22(28.6)	0.29	0.08	1.05	0.060

**Table 2:** Association between early growth and maternal demographics of preterm infants

	Early growth of preterm infants (n = 106)		OR	95% Confidence Interval		P value
	Deficit	Optimal				
**Maternal age**						
< 20 years	8(27.6)	10(13.0)	1			
20-29 years	17(58.6)	49(63.6)	0.43	0.15	1.28	0.13
30-39 years	4(13.8)	15(19.5)	0.33	0.08	1.41	0.136
40-49 years	0(0.0)	3(3.9)	NA	-	-	-
**Marital status**						
Single	13(44.8)	21(27.3)	1.00			
Married	16(55.2)	55(71.4)	0.47	0.19	1.14	0.096
**Education**						
None	4(13.8)	3(3.9)	1.00			
Primary	18(62.1)	53(68.8)	0.25	0.05	1.25	0.092
Secondary	6(20.7)	16(20.8)	0.28	0.05	1.65	0.159
Tertiary	1(3.4)	5(6.5)	0.15	0.01	2.05	0.155

## Discussion

Contrary to what was expected, the preterm infants who were born between 33 - 36 weeks gestational age at birth were at more risk of developing growth deficit than those between 28 - 32 weeks. All the infants who were born less than 28 weeks gestation had optimal growth. This does not agree with a study that was carried in Brazil that showed that extremely low birth weight infants were at more risk of developing complications of prematurity. Infants born between 33 - 36 weeks have also been thought to require less support than those who are born at 32 weeks and below. Since the infants who were born less than 28 weeks gestation had longer hospital stay and better outcomes, it would be necessary to keep the late preterm infants for a longer period in the hospital before discharge. This will empower their guardians with the knowledge to care for the small babies by allowing more time with the health care providers. Follow up of the infants provided very important information in regards to their early growth which was often overlooked during their outpatient visits. Early detection of any deviation in their early growth would greatly assist in timely intervention for better future outcomes of these infants. In support to two studies which were carried out in Kenya and Uganda, most of the preterm infants did not attain the recommended daily weight gain. This could be associated with the many difficulties which were experienced by the guardians during the transition period from the health facility to their homes.

Majority of the mothers were between the ages of 20-29 years (73.9%). This age group has been associated with better survival and outcomes of preterm babies which is a very positive maternal characteristic in this population. Optimal growth was demonstrated in infants whose mothers had any level of literacy in comparison to mothers who had not attained any formal education. Education always empowers the mothers in all aspects and should therefore be emphasized. Mothers who lack or have very low level of literacy should therefore be given extra support by either discharging their infants at a much higher weight or being given one week postnatal return date instead of two. Being married and staying home as a house wife was also shown to contribute positively towards the growth of the infants. A mother who receives family support from a spouse is more likely to be emotionally and psychologically stable which would in turn promote her parenting skills. Those mothers who were housewives could be able to offer their total attention to their infants and hence their better outcome.

This study filled a gap which was identified in prior studies which stated that few studies were carried out in Africa during the postnatal periods of these infants. There was need for continued support of the preterm infants and especially during their immediate post discharge period in the community. This can be facilitated by linking them to level 2 and 3 facilities where their body weight, body length and head circumference can be monitored on weekly basis. However, the study did not follow the infants for a longer duration which would have established the later growth and developmental outcomes of these infants.

## Conclusion

Most of the preterm infants at discharge were females who were born between 33 and 36 weeks gestation. Growth deficit was present in the majority and gestational age at birth was a major determinant of the early growth in these preterm infants. There's need for further research in order to establish the later outcomes of preterm infants after hospital discharge at the Kitui District Hospital.

### What is known about this topic

- Early growth in preterm infants is a predictor of their later growth and developmental outcomes;
- The extremely preterm infants are at increased risk of developing complications of prematurity such as deficit in growth;
- The growth of preterm infants is influenced by different infant and maternal characteristics.

### What this study adds

- This study described the early growth of the preterm infants during their immediate postnatal period which is usually a difficult transition period for both the infant and the guardian;
- It also identified the infant and maternal characteristics that influenced their growth.
